# A novel self-assembling peptide as new submucosal injection solution in endoscopic submucosal dissection

**DOI:** 10.1007/s00464-025-11655-y

**Published:** 2025-03-20

**Authors:** Kenichiro Okimoto, Tomoaki Matsumura, Tsubasa Ishikawa, Shohei Mukai, Satsuki Takahashi, Ryosuke Horio, Chihiro Goto, Akane Kurosugi, Michiko Sonoda, Tatsuya Kaneko, Yuki Ohta, Takashi Taida, Keisuke Matsusaka, Jun Kato, Jun-ichiro Ikeda, Naoya Kato

**Affiliations:** 1 Department of Gastroenterology, Graduate School of Medicine, Chiba University, Inohana 1-8-1, Chiba, 260-8670 Japan; 2 Department of Pathology, Chiba University Hospital, Chiba, Japan; 3 Department of Diagnostic Pathology, Graduate School of Medicine, Chiba University, Chiba, Japan

**Keywords:** ESD, PuraLift, MucoUp

## Abstract

**Background:**

This study evaluates the effectiveness of PuraLift, a novel self-assembling peptide-based submucosal injection solution, in endoscopic submucosal dissection (ESD) procedures. We compared its performance to MucoUp in a variety of organ-spanning lesions (esophagus, stomach, and colon/rectum).

**Methods:**

We included 40 consecutive ESD lesions from our hospital, with 19 treated using PuraLift and 21 using MucoUp. Special cases (such as those with ulcerative colitis, evident fibrosis due to post-treatment scars, and circumferential esophageal cases) and the cases that used device without waterjet function were excluded. Endoscopists assessed the satisfaction of submucosal lifting through needle injection on a 5-point scale. Firmness during local injection by the assistant for the PuraLift group was compared to MucoUp (MucoUp was set as a baseline score of 3) using a 5-point scale.

**Results:**

The firmness during local injection was significantly lower with PuraLift compared to MucoUp across all locations: esophagus (1 (1–2) vs. 3 (3–3), *p* = 0.018), stomach (1.5 (1–2) vs. 3 (3–3), *p* < 0.001), and colon/rectum (2 (1–2) vs. 3 (3–3), *p* < 0.001). However, there were no significant differences between PuraLift and MucoUp in terms of endoscopist satisfaction with lifting, amount of solution injected, glycerol used via jet function, or procedure time for any organ.

**Conclusion:**

PuraLift, with its novel mechanism, offers comparable lifting satisfaction to MucoUp but with less firmness during injection. It presents a promising alternative as a local injection solution in ESD procedures.

Endoscopic submucosal dissection (ESD) is a highly curative treatment widely performed for early cancers or adenomas in the esophagus, stomach, and colon [1–6]. Submucosal (SM) injection is a critical step during ESD to facilitate a good lift and create a prolonged and sustained SM cushion. Various SM injection solutions containing normal saline have been reported to be effective for endoscopic resection. Particularly, viscous solutions are essential for safe treatment [7–16].

Though it is still unclear which SM injection solution is optimal for ESD, MucoUp (Boston Scientific, Marlborough, MA, USA) and Ksmart (Olympus, Tokyo, Japan), based on sodium hyaluronate (HA), are widely used. In our hospital, MucoUp was primarily used for ESD procedures as well.

Recently, a novel SM injection solution called PuraLift has been introduced (3D-Matrix Europe Ltd, Tokyo, Japan). PuraLift is supplied in a 20-ml vial and is recommended for use without dilution. It differs from other viscous solutions in its unique characteristics. PuraLift is a peptide aqueous solution in vials, primarily composed of a group of peptides known as a self-assembling peptide [17, 18]. When exposed to bodily fluids, the peptide solution swiftly undergoes gelation under physiological conditions, such as pH changes or metal ions present, thereby serving as a submucosal lifting agent.

However, the usefulness of PuraLift in ESD has not yet been fully analyzed. Thus, this study aims to investigate the effectiveness of PuraLift in organ-spanning ESD procedures.

## Materials and methods

### Study design

This retrospective study was performed according to the Declaration of Helsinki. The study protocol was reviewed and approved by the Ethics Review Committee of Chiba University Graduate School of Medicine (HK202404-05). We compared two groups: one using PuraLift and the other using MucoUp regarding endopoints.

### Patients and lesions

**Fig. 1:**
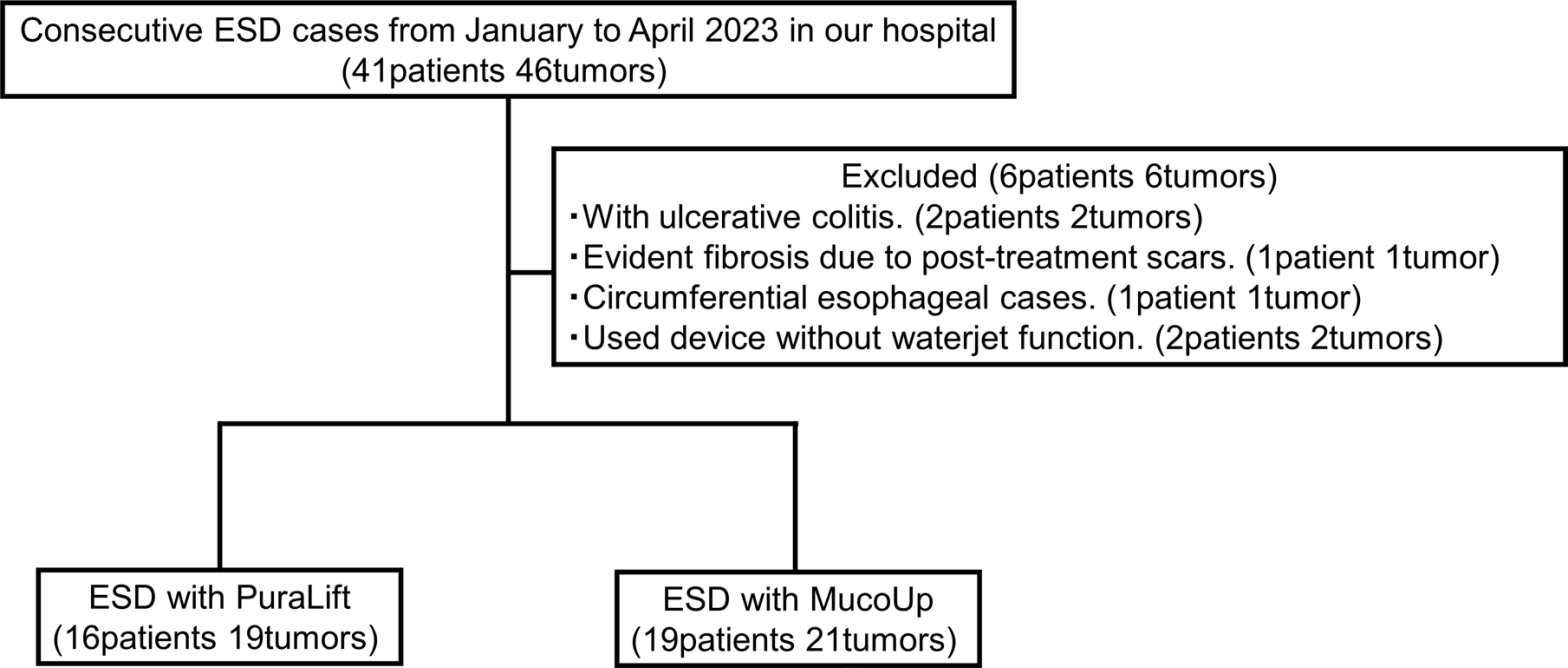
Study flow. *ESD* endoscopic submucosal dissection

Consecutive ESD cases excluding special cases (such as those with ulcerative colitis, evident fibrosis due to post-treatment scars, and circumferential esophageal cases) and the cases that used device without waterjet function such as IT knife 2 (Olympus) at our hospital from January to April 2023 were included, involving the esophagus, stomach, and colon/rectum (Fig. 1).

### Details of use and evaluation for local injection

We primarily used PuraLift in the first half of the period and switched to MucoUp in the latter half, rather than selecting the local injection based on the case. The group that used PuraLift did not use MucoUp and vice versa. We compared the amount of MucoUp or PuraLift used, along with the quantity of 10% glycerin with 0.9% NaCl plus 5% fructose solution (Glycerol; Chugai Pharmaceutical Co, Tokyo, Japan) administered via device-guided local injection, considering lesions backgrounds. MucoUp and PuraLift were used in their undiluted forms. Satisfaction regarding the SM lifting by the endoscopist through needle injection (MucoUp or PuraLift) was assessed on a 5-point scale: 1. dissatisfied, 2. somewhat dissatisfied, 3. neutral, 4. somewhat satisfied, and 5. satisfied. For the group using PuraLift, the firmness during local injection by the assistant compared to the use of MucoUp (Mucoup was set as score 3) was evaluated on a 5-point scale: 1. no resistance, 2. almost no resistance, 3. same, 4. slight resistance, and 5. resistance.

### ESD

ESD was performed mainly using GIF-H290T (for esophagus and stomach) or PCF-H290T (for colon and rectum) (Olympus). For SM injection, after the injection of normal saline into the submucosa, PuraLift or MucoUp with indigo carmine was injected into the properly elevated submucosa using 26G needle (NeedleMaster; Olympus). Dual Knife J 1.5 mm (for esophagus, colon and rectum) or 2.0 mm (for stomach) (Olympus) was used for mucosal incision and SM dissection. During dissection, we used the device’s jet function with glycerol as well. The clip-and-line method was used at the discretion of the endoscopists [19]. The procedure time was defined from the start of submucosal injection to specimen removal.

### Pathological diagnosis

The resected specimens were stretched, pinned flat on a cork board, and then placed in a 10% formalin container. All the tissue samples were stained with standard hematoxylin and eosin and evaluated by pathologists. If the lesion was resected en bloc with lateral and vertical margins were tumor negative, the specimens were defined as “R0 resection specimens.”

### Adverse events

The delayed bleeding was defined as having hematemesis, melena (for esophagus and stomach ESD), or hematochezia or need for blood transfusion, or a decreased hemoglobin concentration of > 2 g/dL after the procedure. Delayed perforation was defined as no perforation during the procedure and no symptoms after resection, with a subsequent sudden appearance of abdominal pain with peritoneal or retroperitoneal free air on computed tomography.

### Endpoint

The primary endpoint was satisfaction regarding submucosal lifting by the endoscopist through needle injection for PuraLift and MucoUp, and the firmness of PuraLift during local injection by the assistant compared to the use of MucoUp (Mucoup was set as score 3) assessed on a 5-point scale. The secondary endpoint included procedure time, amount of submucosal injection, and the amount injected glycerol using the device’s jet function.

### Statistical analysis

Baseline data were presented as *n* (%) or medians with ranges. Data were analyzed using the Mann–Whitney *U*, Chi-squared, or Fisher’s exact test. All statistical analyses were performed using the Statistical Package for the Social Sciences software version 26 (SPSS, Chicago, IL, USA). *p* values of < 0.05 were considered statistically significant.

## Results

### Tumor characteristics

In total, the PuraLift group had 16 patients with 19 tumors, while the MucoUp group had 19 patients with 21 tumors. There were no significant differences observed in the proportions of tumors in the esophagus, stomach, and colon/rectum between the PuraLift and MucoUp groups (*p* = 0.648, Chi-squared test). Additionally, when analyzed by organ-specific factors (tumor size, location, morphology, and histology), no significant differences were found other than the location of the stomach (Table 1).

**Table 1:** Tumors characteristics

Overall	PuraLift (*n* = 19)	MucoUp (*n* = 21)	*p* value
Organ, * n* (%)			
Esophagus	3 (15.8)	4 (19.0)	0.648^a^
Stomach	10 (52.6)	8 (38.1)	
Colorectal	6 (31.6)	9 (42.9)	

Esophagus	PuraLift (*n* = 3)	MucoUp (*n* = 4)	*p* value
Tumor size (mm), median (range)	12 (10–40)	11 (10–20)	0.578^b^
Location (middle/ lower thoracic esophagus)	3/0	3/1	1.000^c^
Morphology (elevated/ others)	0/3	1/3	1.000^c^
Histology (SCC/ Barrett’s adenocarcinoma)	3/0	3/1	1.000^c^

Stomach	PuraLift (*n* = 10)	MucoUp (*n* = 8)	*p* value
Tumor size (mm), median (range)	15 (3–40)	12.5 (8–30)	0,857^b^
Location (U/ M/ L)	2/1/7	3/4/1	0.042^a^
Morphology (elevated/ others)	7/3	3/5	0.184^c^
Histology (adenoma/ IMC/ SM cancer)	0/10/0	1/6/1	0.245^a^

Colon/rectum	PuraLift (*n* = 6)	MucoUp (*n* = 9)	*p* value
Tumor size (mm), median (range)	37.5 (30–70)	35 (20–45)	0.170^b^
Location (right/ left)	3/3	5/4	0.622^c^
Morphology (elevated/ others)	6/0	8/1	1.000^c^
Histology (adenoma/ IMC/ SM cancer)	3/2/1	2/5/2	0.530^a^

*SCC* squamous cell carcinoma, *IMC* intramucosal carcinoma, *SM* submucosa

^a^Chi-squared test

^b^Mann–Whitney *U* test

^c^Fisher’s exact test

### Treatment outcomes

**Fig. 2:**
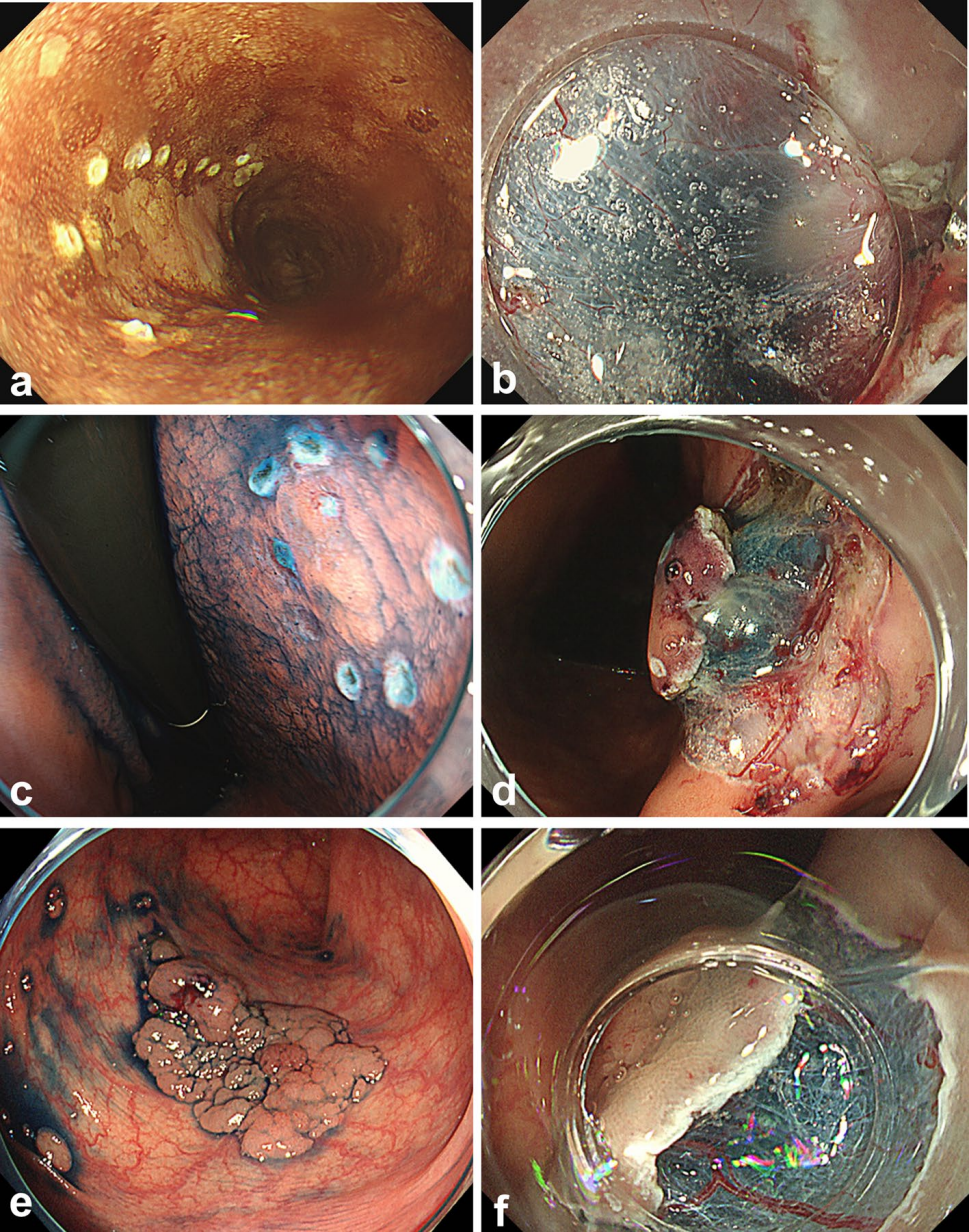
The typical images of esophagus, stomach, and colon treated with PuraLift via needle injection. **a** and **b** Esophagus, **c** and **d** stomach, **e** and **f** colon are the same cases. **a** Esophageal squamous cell carcinoma (12 mm, IIb). The pathological finding was EP Ly0, V0, HM0, and VM0. **b** Right after submucosal injection of PutaLift with indigo carmine. **c** Stomach adenocarcinoma (lesser curvature: 10 mm IIa). The pathological finding was tub1, Ly0, V0, HM0, and VM0. **d** Right after submucosal injection of PutaLift with indigo carmine. **e** Sigmoid colon adenoma (30 mm IIa). The pathological finding was adenoma, HM0, and VM0. **f** Right after submucosal injection of PutaLift with indigo carmine

**Fig. 3:**
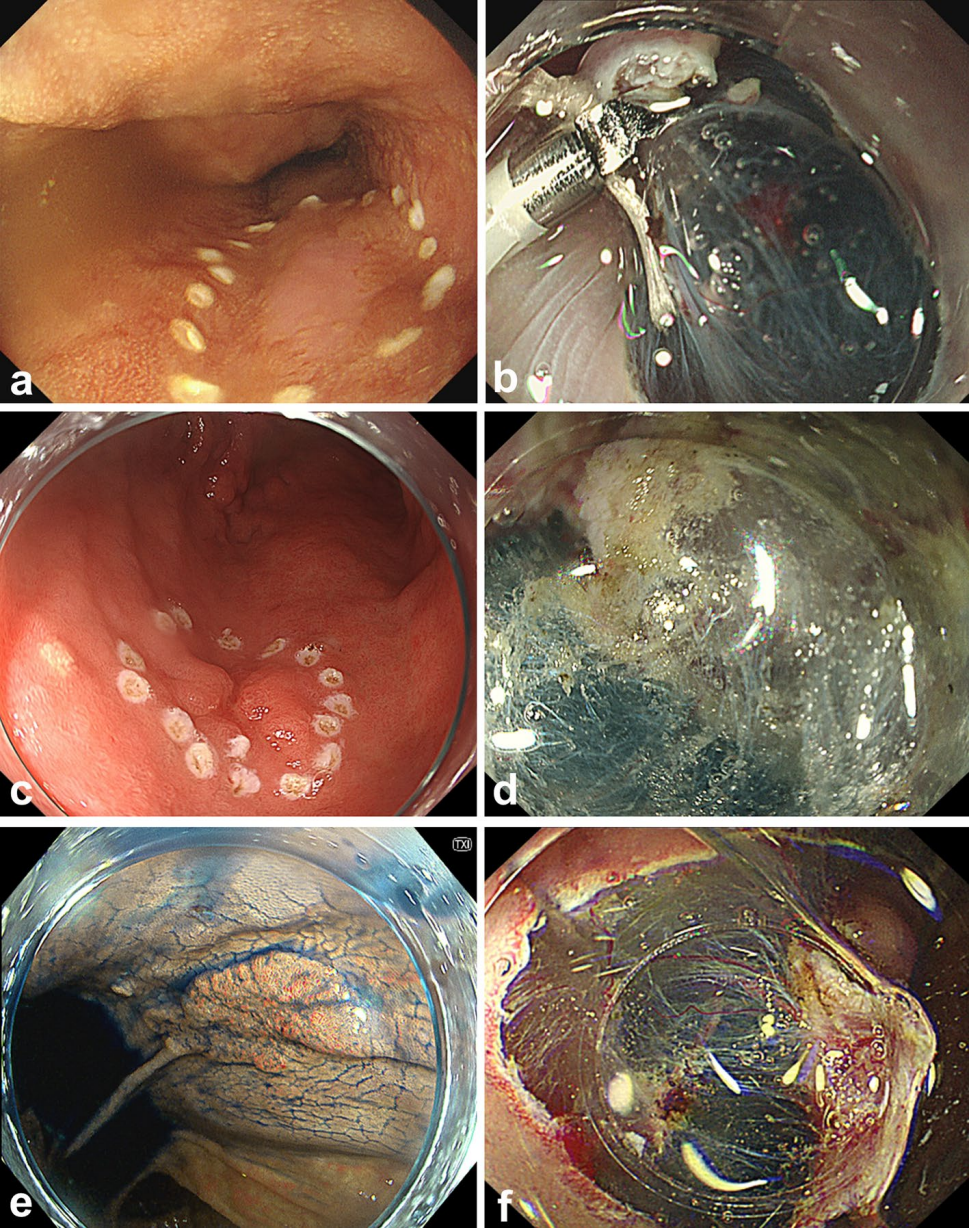
The typical images of esophagus, stomach, and colon treated with MucoUp via needle injection. **a** and **b** Esophagus, **c** and **d** stomach, **e** and **f** colon are the same cases. **a** Esophageal squamous cell carcinoma (12 mm, IIb). The pathological finding was EP Ly0, V0, HM0, and VM0. **b** Right after submucosal injection of MucoUp with indigo carmine. **c** Stomach adenocarcinoma (greater curvature. 10 mm IIa + IIc). The pathological finding was tub1, Ly0, V0, HM0, and VM0. **d** Right after submucosal injection of MucoUp with indigo carmine. **e** Transverse colon adenocarcinoma (25 mm IIa). The pathological finding was tub1, Ly0, V0, HM0, and VM0. **f** Right after submucosal injection of MucoUp with indigo carmine

The typical images of esophagus, stomach, and colon/rectum treated with PuraLift and MucoUp via needle injection are depicted in Figs. 2 and 3, respectively. In addition, treatment outcomes are shown in Table 2. Severe fibrosis was not observed in either group for any organ during ESD, nor were intraoperative/delayed perforations or delayed bleeding observed. Both the PuraLift group and the MucoUp group achieved a 100% en bloc resection rate and a high R0 resection rate ≥ 80% across all organs. Additionally, there were no significant differences between the two groups in the use of the clip-and-line method or in procedure time.

**Table 2:** Treatment outcomes

Overall	PuraLift (*n* = 19)	MucoUp (*n* = 21)	*p* value
Severe fibrosis observed during ESD, * n* (%)	0 (0)	0 (0)	NA
Adverse events related to ESD, * n* (%)			
Intraoperative or delayed perforation	0 (0)	0 (0)	NA
Delayed bleeding	0 (0)	0 (0)	NA

Esophagus	PuraLift (*n* = 3)	MucoUp (*n* = 4)	*p* value
Type of resection, * n* (%)			
En bloc	3 (100)	4 (100)	NA
R0 resection	3 (100)	4 (100)	NA
Use of clip-and-line method, * n* (%)	3 (100)	3 (75.0)	1.000^a^
Procedure time (min), median (range)	50 (20–60)	42.5 (20–65)	0.858^b^

Stomach	PuraLift (*n* = 10)	MucoUp (*n* = 8)	*p* value
Type of resection, * n* (%)			
En bloc	10 (100)	8 (100)	NA
R0 resection	8 (80.0)	8 (100)	0.477^a^
Use of clip-and-line method, * n* (%)	3 (30.0)	6 (75.0)	0.153^a^
Procedure time (min), median (range)	59.5 (30–180)	82 (15–218)	0.594^b^

Colon/rectum	PuraLift (*n* = 6)	MucoUp (*n* = 9)	*p* value
Type of resection, * n* (%)			
En bloc	6 (100)	9 (100)	NA
R0 resection	6 (100)	8 (88.9)	1.000^a^
Use of clip-and-line method, * n* (%)	0 (0)	1 (11.1)	1.000^a^
Procedure time (min), median (range)	67 (46–225)	46 (17–150)	0.098^b^

*NA* not applicable

^a^Fisher’s exact test

^b^Mann–Whitney *U* test

### Factors regarding injection

Factors regarding injection is shown in Table 3. The firmness during local injection by the assistant was significantly lower with PuraLift compared to MucoUp across all locations: esophagus (1 (1–2) vs. 3 (3–3), *p* = 0.018), stomach (1.5 (1–2) vs. 3 (3–3), *p* < 0.001), and colon/rectum (2 (1–2) vs. 3 (3–3), *p* < 0.001). On the other hand, there were no significant differences between the PuraLift and MucoUp groups in terms of satisfaction regarding lifting by the endoscopist although local injection, amount of solution injected via needle, or amount of glycerol via the jet function of the device for any of the organs.

**Table 3:** Factors regarding injection

Esophagus	PuraLift (*n* = 3)	MucoUp (*n* = 4)	*p* value^c^
Satisfaction regarding the lifting^a^	3 (3–3)	3 (3–4)	0.386
The firmness during local injection^b^	1 (1–2)	3 (3–3)	0.018
Amount of solution via injection needle (ml)	11 (10–35)	15 (10–30)	0.714
Amount of glycerol via jet function of device (ml)	80 (30–120)	50 (30–80)	0.467

Stomach	PuraLift (*n* = 10)	MucoUp (*n* = 8)	*p* value
Satisfaction regarding the lifting^a^	3 (3–4)	3 (3–4)	0.388
The firmness during local injection^b^	1.5 (1–2)	3 (3–3)	< 0.001
Amount of solution via injection needle (ml)	20 (10–100)	22.5 (10–65)	0.858
Amount of glycerol via jet function of device (ml)	65 (10–200)	75 (5–160)	0.964

Colon/rectum	PuraLift (*n* = 6)	MucoUp (*n* = 9)	*p* value
Satisfaction regarding the lifting^a^	3.5 (2–4)	3 (3–4)	0.586
The firmness during local injection^b^	2 (1–2)	3 (3–3)	< 0.001
Amount of solution via injection needle (ml)	20 (13–56)	12 (7–65)	0.124
Amount of glycerol via jet function of device (ml)	77.5 (40–150)	60 (10–180)	0.512

All values are expressed using median and range

^a^Satisfaction regarding the lifting by the endoscopist through local injection (MucoUp or PuraLift) was assessed on a 5-point scale: 1. Dissatisfied, 2. Somewhat dissatisfied, 3. Neutral, 4. Somewhat satisfied, and 5. Satisfied

^b^The firmness during local injection by the assistant compared to the use of MucoUp (Mucoup was set as score 3) was evaluated on 5-point scale: 1. No resistance, 2. Almost no resistance, 3. Same, 4. Slight resistance, and 5. Resistance

^c^*p* values are analyzed with Mann–Whitney *U* test

## Discussion

This study is the first paper to verify the effectiveness of PuraLift for ESD across different organs. PuraLift received high satisfaction from endoscopists, and it was possible to inject it with minimal force with almost no stiffness. In conventional ESD, it was perceived to provide at least an equivalent user experience to HA. This product is approved in Japan and undergoing test marketing there, but it remains unapproved in overseas markets. In this study, we evaluated general usability excluding special conditions. To standardize the local injection conditions, we analyzed cases using the Dualknife J with jet function, a device commonly used in our facility and excluded cases using the ITknife 2 without jet function.

MucoUp and Ksmart both have a HA concentration of 0.4% [11]. In this study, evaluation was conducted exclusively on MucoUp, while awaiting future evaluations for Ksmart. However, the report indicates that both MucoUp and Ksmart exhibit higher injection pressures compared to saline, with Ksmart showing significantly higher pressures than MucoUp [11]. During ESD, it is crucial to assess the firmness of the injection to confirm that the injection solution has appropriately reached the SM layer rather than intramuscular injection. When injecting with force, it can be difficult to notice the firmness indicative of inadvertent injection into the muscular layer. It has also been reported that when the puncture is too deep, piercing the muscular layer or serosa, appropriate distension may not be achieved, and patients may experience pain [20]. In this regard, PuraLift is considered to have higher safety.

In addition to HA, commonly used injection solution includes normal saline [21] and glycerol [22]. When injected into the SM layer, HA has been observed to provide greater mucosal elevation compared to these alternatives in porcine stomach models, with similar conditions observed up to 60-min post-injection [23]. In another study, PuraLift and HA tended to maintain height and volume when compared to normal saline in the stomach and colon of living dog [17]. In this study, there was no significant difference in endoscopist satisfaction regarding lifting between PuraLift and MucoUp. PuraLift was considered to provide better SM elevation compared to normal saline and glycerol in this context.

On the other hand, the reason PuraLift exhibits low injection firmness is because it actually undergoes gelation upon contact with bodily fluids after injection. Therefore, during injection, it is initially a low-viscosity fluid, allowing for smooth injection with minimal force. Once it undergoes gelation, it is expected to remain in the SM layer, providing effective lifting. Another product that employs a similar mechanism of self-assembling peptides is PuraStat (3D-Matrix Europe Ltd). PuraStat functions by becoming gelation upon contact with bodily fluids, thereby providing hemostatic effects [24–26]. Additionally, it has been reported to promote ulcer healing and prevent stenosis [27, 28]. The basic mechanism of PuraStat and PuraLift is similar. However, PuraLift has a lower peptide concentration than PuraStat, resulting in a finer mesh structure and a softer gel compared to PuraStat. The effects of local injection of PuraLift on hemostasis and the promotion of ulcer healing after ESD remain to be investigated in future studies.

There are some limitations in this study. First, this is a single-center retrospective study with a small number of cases. Second, clear fibrosis and cases involving circumferential esophagus have been excluded from the analysis. Third, while the cases are broadly categorized by period, the backgrounds are not completely matched. Additionally, ex vivo distension tests were not conducted. Fourth the interventionist such as the endoscopist or assistant knew of which SM injection solution is being used. Fifth, when analyzed by organ, many endoscopists were common between the PuraLift and MucoUp groups, but not all were identical. Sixth, we did not conduct an objective evaluation of submucosal elevation across specific organ and specific colonic segments.

In conclusion, PuraLift is a local injection solution with a novel mechanism, providing comparable satisfaction regarding lifting similar to HA by the endoscopist. Additionally, PuraLift exhibited less firmness with injection compared to HA. PuraLift is expected to be a promising option as new local injection solution in ESD.

## Data Availability

The authors confirm that the data supporting the findings of this study are available within the article.

## Abbreviations

ESD: Endoscopic submucosal dissection
SM: Submucosal
HA: Sodium hyaluronate
NA: Not applicable
